# Characterizing alternative feeds for rainbow trout (*O. mykiss*) by ^1^H NMR metabolomics

**DOI:** 10.1007/s11306-018-1454-5

**Published:** 2018-11-27

**Authors:** Simon Roques, Catherine Deborde, Nadège Richard, Luce Sergent, Francis Kurz, Sandrine Skiba-Cassy, Benoît Fauconneau, Annick Moing

**Affiliations:** 1 INRA, Univ Pau & Pays Adour, E2S UPPA, UMR 1419, Nutrition Métabolisme, Aquaculture, 64310 Saint Pée sur Nivelle, France; 2 Phileo Lesaffre Animal Care, 59700 Marcq-en-Baroeul, France; 3 Bordeaux Metabolome Facility, MetaboHUB, CGFB, Centre INRA de Nouvelle Aquitaine Bordeaux, 33140 Villenave d’Ornon, France; 4 INRA, Univ. Bordeaux, UMR 1332 Fruit Biology and Pathology, Centre INRA de Nouvelle Aquitaine Bordeaux, 33140 Villenave d’Ornon, France; 5 Copalis Industrie, 62480 Le Portel, France; 6 Algae Natural Food, 67400 Illkirch-Graffenstaden, France

**Keywords:** Feed, Metabolomics, Aquaculture, Insect, Microalgae, Yeast

## Abstract

**Introduction:**

Fish feed formulations are constantly evolving to improve the quality of diets for farmed fish and to ensure the sustainability of the aquaculture sector. Nowadays, insect, microalgae and yeast are feedstuff candidates for new feeds. However, the characterization of aquafeed is still based on proximate and targeted analyses which may not be sufficient to assess feed quality.

**Objectives:**

Our aim was to highlight the soluble compounds that specifically differ between selected plant-based feeds complemented with alternative feedstuffs and discuss their origin and potential for fish nutrition.

**Methods:**

A growth trial was carried out to evaluate growth performances and feed conversion ratios of fish fed plant-based, commercial, insect, spirulina and yeast feeds. ^1^H NMR metabolomics profiling of each feed was performed using a CPMG sequence on polar extracts. Spectra were processed, and data were analyzed using multivariate and univariate analyses to compare alternative feeds to a plant-based feed.

**Results:**

Fish fed insect or yeast feed showed the best growth performances associated with the lowest feed conversion ratios compared to plant-based feed. Soluble compound ^1^H NMR profiles of insect and spirulina alternative feeds differed significantly from the plant-based one that clustered with yeast feed. In insect and spirulina feeds, specific differences compared to plant-based feed concerned glycerol and 3-hydroxybutyrate, respectively.

**Conclusion:**

This strategy based on compositional differences between plant-based and alternative feeds can be useful for detecting compounds unsuspected until now that could impact fish metabolism.

**Electronic supplementary material:**

The online version of this article (10.1007/s11306-018-1454-5) contains supplementary material, which is available to authorized users.

## Introduction

The global aquaculture sector has developed constantly over the last two decades (FAO 2016). Fish feed has evolved considerably to support such trends, moving progressively from traditional fish meal (FM) and fish oil (FO) based feeds to plant-based (PB) feeds. The difficulty of supplying all aquaculture sectors with marine resources has been the main reason for replacing FM, which is now largely substituted by cereals and grain legumes (National Research Council (U.S.) 2011). In salmonids, including rainbow trout, FO can be substituted almost exclusively by vegetable oil, which provides essential fatty acid precursors of the long chain polyunsaturated fatty acids docosahexaenoic acid (DHA) and eicosapentaenoic acid (EPA) (Oliva-Teles et al. 2015). The total replacement of FM for carnivorous species by PB feedstuffs, however, leads to reduced growth performances, mainly due to numerous anti-nutritional factors and unbalanced amino acid profiles (Geay et al. 2011; Collins et al. 2013; Lazzarotto et al. 2018). Furthermore, the exclusive use of PB feedstuffs for aquaculture is no longer sustainable. For example, the land used for PB feedstuff production is the same as that used for other livestock feeds and human nutrition (Troell et al. 2014). Thus, there is an urgent need of alternatives to FM and PB products, either other animal protein feedstuffs such as insects, or micro-organisms such as microalgae and yeast.

The potential for insects to be included in the formulation of animal feeds is gaining importance thanks to their high protein content and well-balanced amino acid profiles (Rumpold and Schluter 2013; van Huis 2013; Barroso et al. 2014; Henry et al. 2015; Barragan-Fonseca et al. 2017). Promising results with insect meals have been obtained in carnivorous species including rainbow trout (St-Hilaire et al. 2007; Sealey et al. 2011; Lock et al. 2016; Stadtlander et al. 2017). Microalgae have mostly been used for DHA and EPA substitution and as feed supplement rather than as FM substitution, yet their nutritional values are promising for aquaculture feeding with high contents of protein and high protein digestibility (Nakagawa and Montgomery 2007; Teimouri et al. 2013). *Spirulina platensis* is commonly described as a microalga although it belongs to the cyanobacteria due to several common characteristics such as a prokaryotic structure and peptidoglycan-based cell walls, as opposed to cellulose in algae (Belay et al. 1996; Spolaore et al. 2006; Becker 2007). Yeast, another micro-organism widely known in animal nutrition to provide high quality nutrients, can also be used as a probiotic and for its immune stimulant properties in the form of yeast cell walls (Ferreira et al. 2010; Ringø et al. 2010; Navarrete and Tovar-Ramrez 2014; Broadway et al. 2015). Indeed, yeast cell walls have mainly been used in fish nutrition for their immune function enhancement but have not yet been tested as a substitute for FM (Robersten et al. 1990; Jørgensen et al. 1993; Gopalakannan and Arul 2010; Refstie et al. 2010; Meena et al. 2013).

Commercial fish feeds are usually characterized by their feedstuff composition and their chemical proximate composition, which indicates the protein, lipid and carbohydrate content, as well as amino acids and fatty acids profile of the feed (Jobling 2016). It is possible to predict the nutritional value of a feed based on its chemical nutritional profile. However, this does not guarantee that feed will be well tolerated by fish because of the possible presence of antinutritional factors or other compounds. Large differences in feedstuff quality, as revealed by their severe impact on fish growth and metabolism, appear to be related to compounds other than macro-nutrients such as anti-nutritional factors and derivative compounds produced by poor processing conditions (Cheng et al. 2016). Thus, the numerous soluble compounds in feeds and feedstuffs originating either from feedstuff itself or from their processing need to be characterized.

Metabolomics is a reliable technique used in the food industry to assess the authenticity and quality of foods (Wishart 2008; Cevallos-Cevallos et al. 2009; Mannina et al. 2012; Astarita and Langridge 2013). Over the last decade, metabolomics approaches have emerged in fish nutrition to assess the impact of alternative feedstuffs and diets on fish metabolism (Kullgren et al. 2010; Schock et al. 2012; Wagner et al. 2014; Gatesoupe et al. 2018). Recent studies have used targeted metabolomics directly on fish feeds combined with metabolomics on fish. Using ^1^H NMR profiling, Cheng et al. (2017) identified specific compounds in a diet containing Baltic Sea FM with mussel meal and intact yeast that impacted salmon metabolic response. Jasour et al. (2017) demonstrated the importance of oxidation products in feather meal feedstuff used as a protein source on amino acid digestibility. Similarly, they showed that biogenic amines present in FM modulate growth performances (Jasour et al. 2018). These findings support the idea that numerous soluble feed compounds account for fish feed quality, although untargeted analyses of feeds are rarely carried out to characterize their profiles.

Using ^1^H NMR metabolomics, we analyzed soluble compounds of selected plant-based feeds supplemented either by insect larvae (*Hermetia illucens*) meal, microalgae (*S. platensis*) biomass or yeast (*Saccharomyces cerevisiae*) protein fraction. A trial was implemented in rainbow trout to investigate the effect of each experimental feed on growth compared with trout fed plant-based feed or FM- and FO-based feed as a reference. Our results show that some of the feeds supplemented with alternative feedstuffs presented higher intensities of specific compounds compared to the plant-based feed. Some of these compounds are discussed for their potential impact on fish metabolism.

## Materials and methods

### Fish experimental design

The experiment was carried out in the INRA experimental facilities (UMR1419 Nutrition, Métabolisme, Aquaculture, Donzacq, France) authorized for animal experimentation by the French veterinary service, which is the competent authority (A 64-495-1). The experiment was in strict accordance with EU legal frameworks concerning the protection of animals used for scientific research (Directive 2010/63/EU) and according to the National Guidelines for Animal Care of the French Ministry of Research (decree n°2013-118, February 1st, 2013). The scientists in charge of the experimentation received training and personal authorization (N°B64 10 005). In agreement with the ethical committee “Comité d’Ethique Aquitaine Poissons Oiseaux” (C2EA-73), the experiment reported here did not need approval by a specific ethical committee since it involved only classical rearing practices with all diets used in the experiment formulated to cover the nutritional requirements of rainbow trout. During the experiment, fish were monitored daily. If any clinical symptoms (i.e. morphological abnormality, restlessness or uncoordinated movements) were observed, fish were sedated by immersion in 10 mg/l benzocaine solution and then euthanized by immersion in a 60 mg/l benzocaine solution (anesthetic overdose) for 3 min.

Fifteen groups of thirty juvenile rainbow trout (*Oncorhynchus mykiss*) were randomly constituted (five diets in triplicates). The mean initial body weight was 48.98 ± 1.00 g (n = 450 fish) and it was not significantly different between the tanks. Fish were reared in 100 L tanks in a flow-through system supplied with natural spring water at 17 ± 1 °C. Fish were fed twice daily until visual satiety. The duration of the experiment was 84 days. Mortality was recorded daily. The total biomass and the amount of feed distributed per tank were recorded every 3 weeks. The mean body weight per tank was calculated by dividing the total biomass by the number of fish alive in the tank. Feed conversion ratio (FCR) per tank was calculated by dividing the biomass gain by the amount of feed distributed.

### Feed description and characterization

Five different feeds were tested: a commercial-like (COM) feed containing FM and FO, a totally plant-based feed (PB) and plant-based feeds with 15% insect meal (INS), spirulina biomass (SPI) or yeast protein fraction (YST), respectively (Table 1). A fraction of plant proteins and vegetable oils were substituted with each alternative feedstuff depending on their composition to get isoenergetic, isolipidic and isoproteic diets (proximate compositions are detailed in Online Resource 1). The insect meal was prepared as for a fish protein concentrate: live larvae were frozen, then successively minced, ground and sieved. The mixture was defatted by centrifugation and the supernatant which contained most of the lipid fraction was removed. The residue which contained the protein-enriched fraction was collected and dried. The spirulina meal was prepared from whole spirulina which was dried then ground. The yeast protein fraction was produced from autolyzed yeast, then separated to obtain the parietal fraction which was further dried. The feeds were produced at the INRA experimental fish facility (Donzacq, France). Pellets were obtained using an extrusion cooking process (CLEXTRAL BC45 extruder with double screw, Firminy, France) with a 44.5 bar pressure and a 52 °C mean temperature then dried at 40 °C with an air flux for a minimum of 2 h. A sample of pellets was collected just after production and stored at − 20 °C until extraction. Approximately 1 g of frozen pellets of each diet was cryo-ground (6750 Freezer/Mill, Spex SamplePrep, Metuchen, USA) before freeze-drying.

**Table 1:** Feed formulation (g/100 g feed) of control feeds (COM, PB) and experimental feeds (INS, SPI, YST)

Ingredients	COM	PB	INS	SPI	YST
Fish meal	21.04				
Fish oil	4.88				
Rich DHA algae meal		6.84	6.84	6.84	6.84
Insect meal			15.00		
Sprirulin biomass				15.00	
Yeast protein fraction					15.00
Processed animal proteins^a^	15.00				
Vegetable oils^b^	14.65	18.1	14.95	18.15	17.2
Plant proteins^c^	42.41	70.40	58.79	55.30	56.15
Rapeseed lecithin		1.00	1.00	1.00	1.00
Monocalcium phosphate		1.20	1.00	1.00	1.30
Phytase		0.02	0.02	0.02	0.02
Lysine 78%	0.39	0.50	0.50	0.84	0.50
dl-Methionine 98%	0.44	0.65	0.61	0.56	0.70
Threonine 98%	0.20	0.20	0.20	0.20	0.20
Vitamin premix	0.25	0.30	0.30	0.30	0.30
Vitamin C monophosphate 35	0.04	0.04	0.04	0.04	0.04
Mineral premix	0.25	0.30	0.30	0.30	0.30
Liquid choline	0.15	0.15	0.15	0.15	0.15
Antioxidant	0.15	0.15	0.15	0.15	0.15
Antifungal	0.15	0.15	0.15	0.15	0.15

All feedstuffs are displayed in g/100 g of feed

^a^Processed animal proteins: blood meal, hydrolyzed feather meal, poultry meal

^b^Vegetable oils: rapeseed oil and linseed oil

^c^Plant proteins: wheat gluten, hydrolyzed wheat gluten, pea protein, fababean protein concentrate, soy concentrate, soybean meal, rapeseed meal, peeled fababean and wheat. Amongst the plant proteins, soybean meal, rapeseed meal and peeled fababean were only used in COM feed. A fraction of wheat gluten, pea protein, soy concentrate and wheat and rapeseed oil and linseed oil were substituted by alternative feedstuffs in experimental feeds

Extraction was performed on five randomized replicates of 50.0 ± 0.5 mg of freeze-dried powder as previously published by Moing et al. (2004). Briefly, lyophilized powdered samples were successively extracted with 2 ml of 80/20 (v/v) ethanol (Absolute, Sigma-Aldrich, St Quentin-Fallavier, France) and MilliQ water (Millipore, Molsheim, France), 2 ml of 50/50 ethanol and MilliQ water and 3 ml of 0/100 ethanol and MilliQ water at 80 °C for 15 min. The supernatants were combined, dried under vacuum (SpeedVac Savant, Asheville, USA) and freeze-dried. Extracts were then solubilized in 300 µl of a deuterated solution [200 mM potassium phosphate buffer solution, 2 mM ethylene diamine tetra-acetic acid disodium salt (EDTA)] at apparent pH 6. EDTA was added to chelate paramagnetic cations in order to improve the spectrum resolution, especially in the citrate area. The pH was adjusted to apparent pH 6.00 ± 0.02 using NaOD or DCl by means of a titration robot (BTpH, Bruker, Kalrsruhe, Germany). Adjusted extracts were frozen and freeze-dried. The dried pH-adjusted extracts were stored in obscurity under vacuum at room temperature until ^1^H NMR analysis. Each extract was solubilized in 600 µl of D_2_O and 10 µl of TSP [(trimethylsilyl)propionic-2,2,3,3-d4 acid sodium salt] 9.5 mM before vortexing and 5 min centrifuging at 17,746×*g* (Juan A14, ThermoFisher Scientific, City, country). Five hundred and fifty microliters were then transferred to a 5 mm NMR tube (Wilmad, Vineland, USA) for NMR acquisition. For NMR analysis, D_2_O (99.8% D) was purchased from Eurisotop (Gif sur Yvette, France), TSP (98%) and EDTA from Sigma-Aldrich.

### NMR acquisition

NMR spectra were acquired on a Bruker Avance III 500 MHz spectrometer (Bruker, Wissembourg, France) operating at 500.16 MHz at 300 K using a 5 mm ATMA broadband inverse probe flushed with nitrogen gas. 1D spectra were acquired using a Carr–Purcell–Meiboom–Gill (CPMG) sequence with a T2 filter to remove macromolecule signals and pre-saturation to remove the water signal. CPMG acquisition sequence does not give quantitative information because it reduces signal intensities of compounds based on their spin–spin (T2) relaxation times, and signal intensity is no longer correlated to absolute concentration only. Moreover, the short recycle delay (2 s) may truncate FID of long spin–lattice (T1) resonances. However, comparisons of a selected bin intensity between samples remained possible as acquisition parameters were kept constant. Sixteen scans of 64K data points were recorded with a 90° pulse angle, a 6000 Hz spectral width, a 5.46 s acquisition time, a 2 s recycle delay, 200 µs echo time and 400 loops, preceded by 16 dummy scans.

The assignments of metabolites in the ^1^H NMR spectra were based on chemical shifts, signal multiplicities, intensity ratios, comparison with database values (HMDB, http://www.hmdb.ca, BMRB, http://www.bmrb.wisc.edu and ChenomX NMR Suite 8.3 library (ChenomX Inc., Edmonton, Canada)) and with spectra of authentic compounds recorded in the same solvent conditions (in-house library) and by spiking the samples with corresponding compound standards when doubt remained. 2D NMR experiments (COSY, Correlation SpetroscopY; HSQC, Heteronuclear Single Quantum Correlation; HMBC, Heteronuclear Multiple Bond Correlation) with non-uniform sampling (NUS percent at 25% and 128 points for COSY and HSQC and 512 points for HMBC) were performed for selected samples for assignment verification. COSY experiment observed ^1^H nuclei in both direct and indirect dimensions where 16 dummy scans preceded a 64-scan series of 8.97 µs 90° pulse in each dimension. Spectral width was 6000 Hz for each dimension, with 4K data points in f2 and 1K in f1. The HSQC experiment observed ^1^H in direct dimension and ^13^C in indirect dimension, 16 dummy scans preceded a 128-scan series of 8.97 µs 90° pulse in direct dimension and 14.50 µs 90° pulse in indirect dimension. Spectral width was 20,000 Hz in indirect dimension and 6,000 in the direct one, 2K data points were recorded in the direct dimension and 1K in the indirect one. The HMBC experiment observed ^1^H in direct dimension and ^13^C in indirect dimension, 16 dummy scans preceded a 128-scan series of 8.50 µs 90° pulse in direct dimension and 14.50 µs 90° pulse in the indirect dimension. Spectral width was 30,000 Hz in indirect dimension and 6000 in the direct one, 4K data points were recorded in the direct dimension and 2K in the indirect one. The 2D spectrum was reconstructed using the recursive multidimensional decomposition available within the Bruker software.

Identification levels were based on the Metabolomics Standards Initiative (Sumner et al. 2007). A representative ^1^H NMR CPMG spectrum of each feed extract has been uploaded to the Zenodo repository (https://zenodo.org/record/1423124; 10.5281/zenodo.1423124).

### Spectra processing

Free induction decay (FID), spectra processing, signal-to-noise ratio determination and spectral data reduction (binning) were processed using the NMRProcFlow tool (Jacob et al. 2017). Prior to Fourier transformation, each FID was zero-filled to 256K data points and multiplied by an exponential window function with a line broadening of 0.3 Hz. Spectra were then automatically zero-order phased and manually calibrated on –CH_3_ groups of TSP-d4 (δ 0 ppm) before manual baseline correction and resonance alignments. Residual water signal (δ 4.768–4.789 ppm) was excluded. Binning was performed by the adaptive intelligent binning method of Meyer et al. (2008) on the spectral area δ 0.896–9.134 ppm. Binning consists in dividing spectra into smaller spectral regions or “bins” to isolate and integrate the area under the signal curve to obtain extracted bin intensities. The total intensity of each spectrum was kept constant and each spectral bin intensity was normalized to this constant sum before exporting the resulting table of relative bin intensities for statistical analyses. Spectral bins with a signal-to-noise ratio under 3 were removed. Thereafter, relative bin intensities will be referred as “relative intensities”.

### Data analyses

Statistical analyses were performed with R statistical software (v.3.3.2, R Development Core Team, 2008) and BioStatFlow web application (v.2.8, http://www.biostatflow.org). To study the effect of feed on fish final body weight and FCR, the three supplemented plant-based feeds were compared to the COM and PB control feeds. First, a one-way ANOVA was carried out on the five feeds to assess their effect on final body weight. Then, the mean fish final body weight or FCR of each experimental feed was compared to the COM or PB control mean by using Student’s *t* test. Differences were considered statistically significant when *P* < 0.05.

For feed analysis, a principal component analysis (PCA) was run on 473 ^1^H NMR spectral bins used as variables for a total of 23 feed samples after removal of one outlier sample in PB and one in COM feed. Variables were Pareto-scaled prior to PCA. The same 473 variables were used for volcano plot analyses with Wilcoxon’s test with false discovery rate (FDR) correction. Signals with significant differences at the *P* < 0.001 level and fold threshold between means set to 2 were considered to highlight the most affected spectral bins.

## Results

### Fish zootechnical performances

Fish final body weight and FCR of juvenile rainbow trout fed control and experimental feeds for 84 days are presented in Table 2. Feeds had a significant impact on final body weight (ANOVA, *P* = 0.023). Fish fed INS and YST feeds had a significantly higher body weight than fish fed PB feed (Student’s *t* test, *P* = 0.004 and *P* = 0.010, respectively). Final body weight of the SPI and COM groups did not significantly differ from that of the PB group (Student’s *t* test, *P* = 0.219 and P = 0.0625, respectively). However, the *P*-value (*P* = 0.065) for the COM group was close to the defined threshold, indicating a trend in higher body weight. Final body weight in the COM group was not significantly different from that of the other groups. FCR differed significantly between the feeds (ANOVA, *P* = 0.002). INS, YST and COM diets were significantly more efficient than PB (Student’s *t* test). On the other hand, SPI feed did not differ from COM or PB feeds.

**Table 2:** Trout body weight for the five feeds after 84 days feeding

	COM	PB	INS	SPI	YST
Initial body weight (g)	49.10 ± 1.01	48.67 ± 1.35	48.90 ± 1.01	48.90 ± 1.01	49.33 ± 1.35
Final body weight (g)	279.10 ± 16.90	247.11 ± 13.99	303.33 ± 8.32*	273.33 ± 27.84	284.44 ± 5.34*
Feed conversion ratio	0.82 ± 0.02*	0.98 ± 0.03^†^	0.86 ± 0.02*^†^	0.92 ± 0.06	0.88 ± 0.02*^†^

Feed conversion ratio (FCR) associated with the five feeds. *COM* commercial, *PB* plant-based, *INS* insect, *SPI* spirulina, *YST* yeast. Mean ± standard deviation (n = 3 tanks)

*Significant difference compared to PB feed according to Student’s *t* test (*P* < 0.05)

^†^Significant difference compared to COM feed according to Student’s *t* test (*P* < 0.05)

### Feed composition overview with PCA

**Fig. 1:**
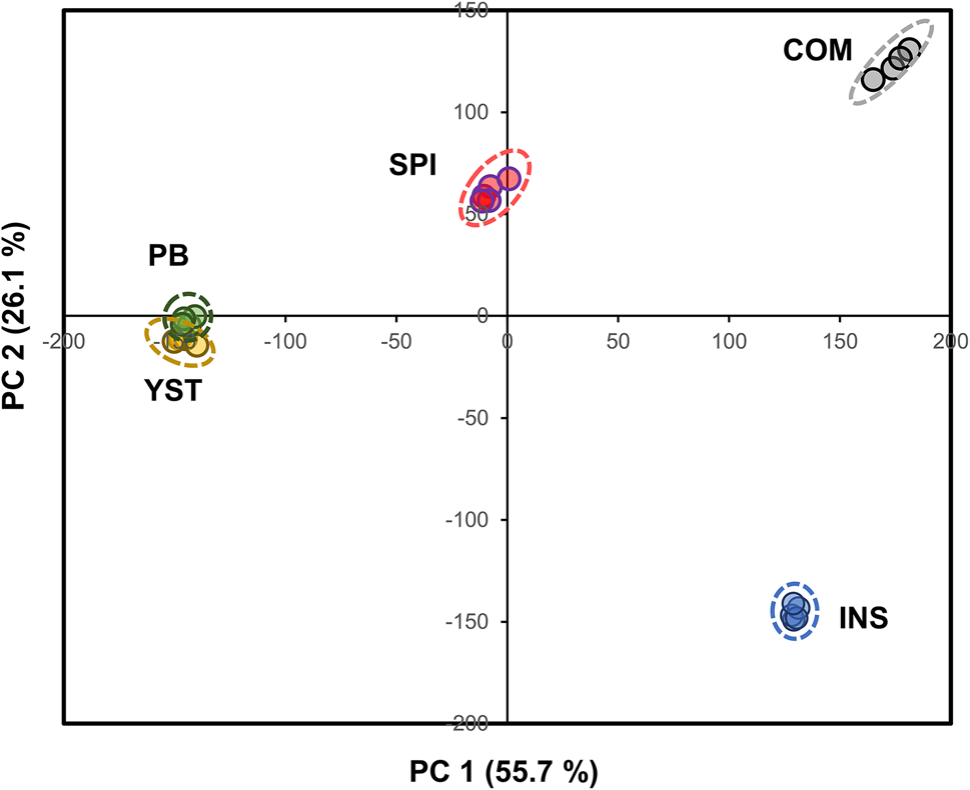
PCA of 473 spectral bins measured in COM, PB, YST, INS and SPI trout feeds using ^1^H NMR profiling of polar extracts. PCA scores plot of first two principal components with four or five replicates per feed. COM, grey; PB, green; YST, yellow; INS, blue; SPI, red symbols

A PCA was performed on the 473 spectral bins of the ^1^H NMR CPMG feed profiles (Fig. 1). The first two principal components (PCs) explained 82% of the total variance. Feed extract samples were well clustered and clearly separated depending on the feedstuff composition, except for YST feed samples which overlapped PB ones. INS and COM feeds were both on the positive side of PC1, compared to PB and YST feeds on the negative side, and they clearly separated along PC2. SPI, PB and COM samples were clearly separated. SPI samples had an intermediary position between PB and COM feeds.

### Comparison of each alternative feed with PB

**Fig. 2:**
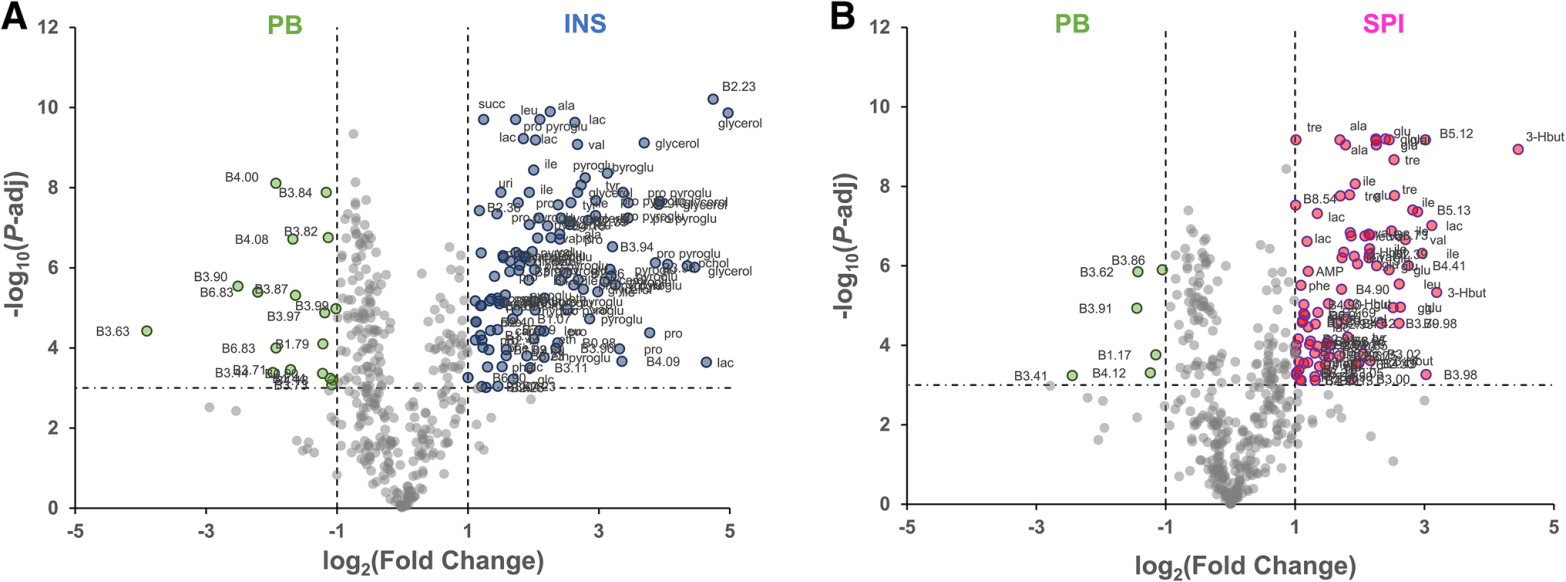
Volcano plot analysis of INS or SPI alternative feeds compared to PB feed for 473 spectral bins measured in feeds using ^1^H NMR profiling of polar extracts. Details with all variable names, annotations, ratios and P-values are presented in Online Resources 3 and 4. **a** Volcano plot analysis of PB and INS feeds with Wilcoxon’s test. Non-annotated dots correspond to metabolite features with adjusted *P* > 0.001 or 0.5 < INS/PB ratio < 2. All features with adjusted *P* < 0.001 and INS/PB ratio < 0.5 or > 2 are annotated. All identified compounds corresponding to these bins are annotated. **b** Volcano plot analysis of PB and SPI feeds with Wilcoxon’s test. Non-annotated dots correspond to metabolite features with adjusted *P* > 0.001 or 0.5 < SPI/PB ratio < 2. All features with adjusted *P* < 0.001 and SPI/PB ratio < 0.5 or > 2 are annotated. All identified compounds corresponding to these features are annotated. *3-Hbut* 3-hydroxybutyrate, *AMP* adenosine mono-phosphate, *ala* alanine, *carn* carnitine, *eth* ethanolamine, *glc* glucose, *glu* glutamate, *hypox* hypoxanthine, *ile* isoleucine, *lac* lactate, *leu* leucine, *orn* ornithine, *phe* phenylalanine, *pchol* phosphocholine, *pro* proline, *pyroglu* pyroglutamate, *succ* succinate, *tyr* tyrosine, *uri* uridine, *val* valine

Volcano plot analyses on the 473 ^1^H NMR CPMG spectral bins of feed extracts were used to assess which spectral regions and corresponding compounds differentiated INS, SPI or YST alternative feeds from the PB control (Fig. 2 and Online Resources 3 and 4). In INS feed (Fig. 2a), 119 ^1^H NMR spectral bins were significantly higher and 19 bins were significantly lower than in PB feed with the chosen thresholds. The highest significant log_2_(FC) reached almost 5 and the lowest was almost − 4. For SPI diet (Fig. 2b), 93 ^1^H NMR spectral bins were significantly higher, whereas 6 bins were significantly lower than in PB feed with the chosen thresholds. The highest significant log_2_(FC) reached about 4.5 and the lowest was about − 2.5. For YST feed (Online Resource 2), only one ^1^H NMR bin was significantly higher, while none was lower than in PB feed. The log_2_(FC) associated of this bin was 1.5. The ^1^H NMR spectral bins highlighted with the volcano plots were tentatively annotated.

**Fig. 3:**
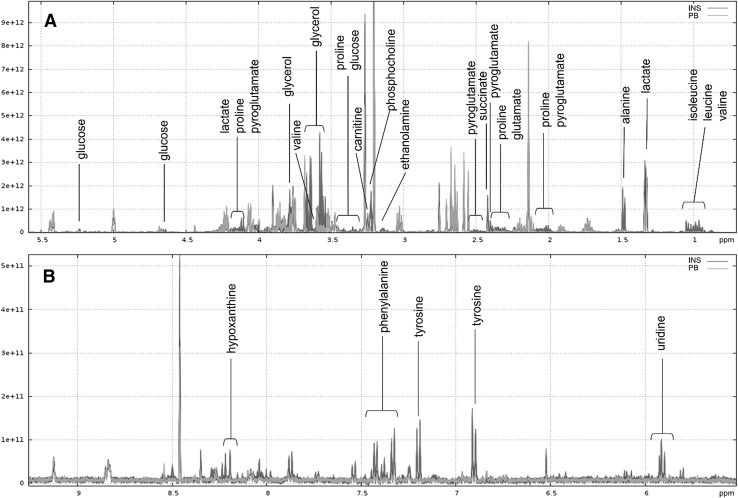
Superimposition of one representative ^1^H NMR CPMG spectrum of a polar extract for INS and one for PB feed. **a** Spectral region δ 0.75–5.5 ppm. **b** Spectral region δ 5.5–9.2 ppm. Annotated compounds correspond to variables highlighted in volcano plots with fold change > 2 and *P*-adj < 0.001. Spectrum of PB feed extract in green and that of INS in blue

**Fig. 4:**
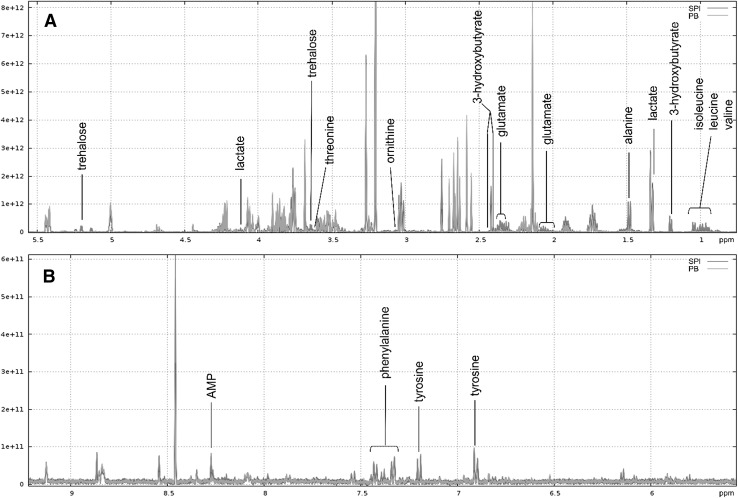
Superimposition of one representative ^1^H NMR CPMG spectrum of a polar extract for SPI and one for PB feed. **a** Spectral region δ 0.75–5.5 ppm. **b** spectral region δ 5.5–9.2 ppm. Annotated compounds correspond to variables highlighted in volcano plots with fold change > 2 and *P*-adj < 0.001. Spectrum of PB feed extract in green and that of SPI in red

Figures 3 and 4 shows annotated superimposition of one representative ^1^H NMR CPMG spectrum for INS or for SPI compared to one representative spectrum of PB feed extract, respectively. Signals were well superimposed within each feed, which demonstrated a good reproducibility. The annotated signals—16 in INS and 12 in SPI—correspond to spectral bins that fulfilled the volcano plot thresholds (Online Resources 3 and 4). INS feed exhibited higher relative intensities of four essential free amino acids—isoleucine, leucine, valine, and phenylalanine—compared to PB feed. Similarly, the relative intensities of free non-essential and other free amino acids, alanine, proline, tyrosine and pyroglutamate, were higher in INS feed compared to PB. The relative intensities of the organic acids lactate and succinate were also higher in INS feed compared to PB. Finally, glucose, glycerol (showing the highest fold change of 31), phosphocholine, ethanolamine, uridine, hypoxanthine and carnitine were identified, and their relative intensities were also higher in INS than in PB feed. We verified the presence of glycerol in the INS feedstuff polar extract, as it presented the highest fold change in the corresponding feed (Online Resource 6A). For SPI feed, the relative intensities of four essential free amino acids, isoleucine, leucine, valine and phenylalanine, were higher than in PB feed. The non-essential free amino acids ornithine, alanine and glutamate, the organic acids lactate, 3-hydroxybutyrate (showing the highest fold change of 22) as well as AMP and trehalose were also identified in SPI feed and their relative intensities were higher than in PB feed. We verified the presence of 3-hydroxybutyrate in an SPI feedstuff polar extract as it presented the highest fold change in the corresponding diet (Online Resource 6B).

## Discussion

The ^1^H NMR metabolomics approach was carried out to explore the composition of new aquaculture feeds compared to a plant-based feed. In the quest for sustainable feed, we focused on INS, YST and SPI. Thanks to an approach based on untargeted feed characterization, numerous free soluble compounds were highlighted. Several of these compounds are known to have an impact on fish metabolism and could thus potentially help to explain differences in growth performances. Growth and FCR were significantly impacted by several alternative diets compared to PB. Our findings indicate that INS or YST feedstuff inclusion in these plant-based feeds can improve growth performances of trout. Insect meal and yeast are already known to be good candidates for FM substitution in rainbow trout and Atlantic salmon (Øverland et al. 2013; Stadtlander et al. 2017). However, the price of each of these feedstuffs is higher than that of fish meal so far, especially for insect meal as insect production chains are still emerging. Our results confirm the interest of complementing PB feeds with insect or yeast feedstuffs.

Similar soluble compounds were found in YST and PB feed. The yeast feedstuff used was a yeast parietal fraction. Yeast cell walls are widely composed of non-soluble polysaccharides, mainly β-glucans, mannan-oligosaccharides and proteins. This probably explains why so few of the soluble compounds of YST and PB feed are significantly different (Lesage and Bussey 2006; Alsteens et al. 2008). Therefore, the higher growth performance of YST feed compared to PB feed cannot be attributed to a direct effect of soluble compounds. However, it could be reasonably ascribed to other demonstrated effects such as changes in gut health and the immune effect of yeast cell walls, which could offset the negative effect of pathogens or enhance nutrient digestibility (Jørgensen et al. 1993; Refstie et al. 2010; Broadway et al. 2015).

On the other hand, differences in the relative intensities of soluble compounds in INS and PB feeds were greater. The insect meal tested contained high quantities of low molecular weight compounds (see molecular weight profile in Online Resource 5). This seems in agreement with the higher relative intensities of several free amino acids found in the INS feed than in the PB feed. Feeding high levels of low molecular weight compounds to carnivorous fish such as salmon or trout has been shown to increase growth (Hevroy et al. 2005; Aksnes et al. 2006). A similar positive impact on growth was also observed with insect meal in the present study performed in rainbow trout. It is known that free amino acids and small peptides are absorbed specifically and more rapidly than the bolus of amino acids produced by protein digestion, especially in juveniles (Oliva-Teles et al. 1999; Lundquist and Artursson 2016; Wang et al. 2017). Furthermore, amino acids, especially branched-chain amino acids, are known as signaling molecules. Together with insulin, branched-chain amino acids, especially leucine, contribute to the activation of the TOR signaling pathway, which regulates protein translation and metabolism (Seiliez et al. 2008; Laplante and Sabatini 2012; Zhang et al. 2017). As the highest relative intensities of free branched-chain amino acids were found in INS feeds, which exhibited the best growth performances, our results support the hypothesis that the positive impact on growth with INS feedstuff may be due to enhanced protein synthesis under amino acid stimulation.

Glycerol relative intensities were higher in INS feed than in PB. Some insect larvae are known to produce glycerol, which protects insects from extreme cold temperatures (Storey and Storey 2012). Moreover, the analysis of the insect raw material used in the present study confirmed that the glycerol originated from this feedstuff. In fish nutrition, glycerol is primarily a substrate of lipid metabolism for the synthesis of triglycerides and phospholipids. However, this substrate is not limiting due to the large dietary supply of lipids. Glycerol seems to act as an energetic substrate in herbivorous species such as Nile tilapia (*Oreochromis niloticus*). However, in carnivorous species, Menton et al. (1986) reported that glycerol is not an effective energy supplier because its conversion into glucose exceeds the glucose requirement of such fish (Neu et al. 2013; da Costa et al. 2015, 2017). Glycerol could act as a microorganism substrate, as *Enterococus* populations are modulated in gut microbiota of rainbow trout fed insect feed (Bruni et al. 2018). In addition, microbiota plays a key role in glycerol degradation in human (Weirdt et al. 2010). However, our data are too limited to support this hypothesis.

The dried spirulina biomass used in the present study contained a complete set of the micro-organism metabolites. Indeed, the higher fold change with SPI feed corresponded to 3-hydroxybutyrate. Like several micro-organisms, *S. platensis* is known to synthesize poly-3-hydroxybutyrate (PHB) and activate depolymerization to use monomers as carbon source under nitrogen-starved conditions (Jau et al. 2005). Moderate inclusion levels of poly (3-hydroxybutyrate) enhance growth in the carnivorous fish European sea bass and Siberian sturgeon, but there are still no data on 3-hydroxybutyrate supplied in the free form as in SPI feed (Schryver et al. 2010; Najdegerami et al. 2012). As a ketone body, 3-hydroxybutyrate could be used as energy substrate in the event of glucose depletion during fasting for specific tissues such as brain and heart in mammals (Newman and Verdin 2017). Moreover, 3-hydroxybutyrate could act as an epimetabolite (Showalter et al. 2017) by inducing lysine histone methylation (Newman and Verdin 2017). However, little is known about its utilization in fish.

Similarly, higher relative intensities of trehalose were detected in SPI compared to PB feed. This higher intensity may directly result from trehalose presence in the SPI feedstuff, and suggests that the spirulina culture conditions may have been sub-optimal as *S. plantensis* is known to produce trehalose under stress (Ohmori et al. 2009). Trehalose in rainbow trout can be hydrolyzed into glucose by brush border trehalase in gastrointestinal mucosa, but the regulation of its enzymatic activity by carbohydrates in diets is unclear as contradictory results were found with other disaccharide enzymes (Buddington and Hilton 1987; Krogdahl et al. 2004). To check this hypothesis, the production of glucose from trehalose should be monitored in trout.

Other mechanisms such as the effect of microbiota or differences in the digestibility of diets are probably involved in the growth performances of alternative feeds. Apparent digestibility of alternative feedstuffs should not be the main cause of growth performance differences as it seems quite high in yeast protein fraction (92.1% of protein, unpublished data) and in published data on similar feeds including spirulina biomass and *H. illucens* larvae meal (Hernández et al. 2012; Magalhães et al. 2017). However, our untargeted approach highlighted several compounds that are rarely described in the analysis of feeds, despite their potential metabolic activity. Their role deserves to be investigated into more detail.

## Conclusions

^1^H NMR metabolomics profiling revealed differences in the soluble compounds of new aquaculture feeds, except for YST feed that could not be discriminated from PB feed due to the low solubility of the cell wall fraction of the yeast feedstuff. Numerous soluble compounds in INS and SPI feeds differed from those in PB feed. Some of these compounds are specific to the insect or to the microalgae. This metabolomic approach to feed characterization could prove relevant in revealing unsuspected compounds that potentially impact fish metabolism. Future trends in the untargeted analysis of aquaculture feeds should also focus on the characterization of alternative feedstuffs themselves to assess their potential values and limits.

## Electronic supplementary material

Below is the link to the electronic supplementary material.

Supplementary material 1 (DOCX 342 KB)

## Data Availability

Representative ^1^H NMR CPMG spectra of each feed extract has been uploaded to the Zenodo repository (https://zenodo.org/record/1423124; 10.5281/zenodo.1423124).

## Abbreviations

COM: Commercial
CPMG: Carr–Purcell–Meiboom–Gill
DM: Dry matter
INS: Insect
FC: Fold change
FCR: Feed conversion ratio
FM: Fish meal
FO: Fish oil
NMR: Nuclear magnetic resonance spectroscopy
PB: Plant-based
PCA: Principal component analysis
SPI: Spirulina
YST: Yeast
