# Supplementary material for: Characterizing alternative feeds for rainbow trout (O. mykiss) by 1H NMR metabolomics
Source: Metabolomics. 2018 Nov 27;14(12):155. doi: 10.1007/s11306-018-1454-5 (PMC6267160; doi:10.1007/s11306-018-1454-5)
Supplement: Supplementary file 1 — Supplementary material 1 (DOCX 342 KB) [file 11306_2018_1454_MOESM1_ESM.docx]

**Characterizing alternative feeds for rainbow trout (*O. mykiss*) by ^1^H-NMR metabolomics**

Simon Roques^1,2,3^, Catherine Deborde^3,4^, Nadège Richard^2^, Luce Sergent^5^, Francis Kurz^6^, Sandrine Skiba-Cassy^1^, Benoît Fauconneau^1^, Annick Moing^3,4^

^1^ INRA, Univ Pau & Pays Adour, E2S UPPA, UMR 1419, Nutrition Métabolisme, Aquaculture, Saint Pée sur Nivelle, F-64310, France

^2^ Phileo Lesaffre Animal Care, 59700 Marcq-en-Baroeul, France

^3^ Bordeaux Metabolome Facility, MetaboHUB, CGFB, Centre INRA de Nouvelle Aquitaine Bordeaux, 33140 Villenave d’Ornon, France

^4^ INRA, Univ. Bordeaux, UMR 1332 Fruit Biology and Pathology, Centre INRA de Nouvelle Aquitaine Bordeaux, 33140 Villenave d’Ornon, France

^5^ Copalis Industrie, 62480 Le Portel, France

^6^ Algae Natural Food, 67400 Illkirch-Graffenstaden, France

*Corresponding author:* simon.roques@inra.fr *tel: +33 5 57-12-24-21*

**Online Resource 1:** Proximate analysis of diets.

|  | COM | PB | INS^a^ | SPI^b^ | YST^c^ |
| --- | --- | --- | --- | --- | --- |
| Dry matter (%) | 95.8 | 96.9 | 95.0 | 96.2 | 97.0 |
| Proteins (% DM) | 43.0 | 44.7 | 42.6 | 45.5 | 44.8 |
| Lipids (% DM) | 21.0 | 21.7 | 22.9 | 20.7 | 19.6 |
| Energy (KJ/g DM) | 24.1 | 24.0 | 24.7 | 23.9 | 23.7 |

^a^insect meal: 51% proteins, 21.3% lipids.

^b^spirulina biomass: 53% proteins, 1.1% lipids

^c^yeast protein fraction: 50% proteins, 8.0% lipids

**Online Resource 2:** Volcano plot analysis of alternative feed YST compared to PB. Volcano plot analysis of PB and YST diets with Wilcoxon’s test for 473 spectral bins measured in diets using 1H-NMR profiling of polar extracts. Non-annotated dots correspond to metabolite features with adjusted P>0.001 or 0.5< INS/PB ratio <2. All features with adjusted P<0.001 and INS/PB ratio <0.5 or >2 are annotated. All identified compounds corresponding to these bins are annotated. Details with all variable names, annotations, ratios and P-values are presented in Online Resource 4 and 5.


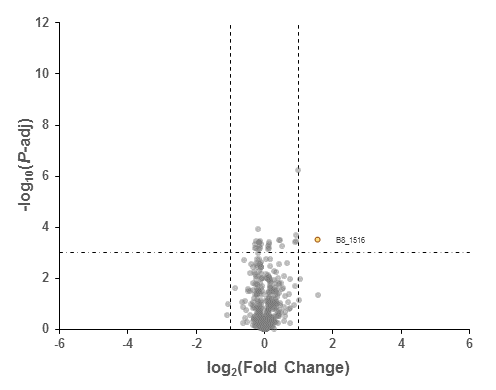


**PB**

**YST**

**Online Resource 3:** Compound table from volcano plots (Fig.2 and Online Resource 3). All signals that fulfil the volcano plot thresholds are annotated by their putative name.

| Spectral bins | Compound signal | FC INS | p-adj INS | Compound signal | FC SPI | p-adj SPI | Compound signal | FC YST | p-adj YST |
| --- | --- | --- | --- | --- | --- | --- | --- | --- | --- |
| B8_5446 |  | 0.63 | 3.84E-06 | Unknown | 2.01 | 2.98E-08 |  | 1.99 | 5.90E-07 |
| B8_2762 |  | 1.10 | 3.75E-01 | Unknown | 2.30 | 1.38E-06 |  | 1.88 | 2.07E-04 |
| B8_2365 | Unknown | 3.00 | 1.57E-04 | Unknown | 2.04 | 4.87E-04 |  | 1.21 | 3.02E-01 |
| B8_2181 | hypoxanthine | 2.79 | 7.86E-06 |  | 1.29 | 1.43E-01 |  | 0.92 | 6.37E-01 |
| B8_1944 | hypoxanthine | 3.37 | 1.17E-05 |  | 1.61 | 4.30E-03 |  | 1.07 | 7.33E-01 |
| B8_1516 |  | 1.00 | 9.52E-01 |  | 1.04 | 4.69E-01 | Unknown | 2.94 | 3.18E-04 |
| B8_0414 | Unknown | 2.32 | 9.24E-04 |  | 0.88 | 2.07E-01 |  | 1.59 | 2.62E-03 |
| B7_4465 | Phenylalanine (phe) | 2.35 | 9.73E-05 | Phenylalanine (phe) | 2.20 | 2.87E-04 |  | 1.08 | 7.28E-01 |
| B7_4312 | Phenylalanine (phe) | 2.74 | 5.74E-06 | Phenylalanine (phe) | 2.13 | 3.11E-06 |  | 0.98 | 7.25E-01 |
| B7_4184 | Phenylalanine (phe) | 2.31 | 8.83E-06 | Phenylalanine (phe) | 1.76 | 1.13E-04 |  | 0.98 | 8.76E-01 |
| B7_3898 | Phenylalanine (phe) | 2.46 | 2.99E-04 | Phenylalanine (phe) | 2.03 | 5.53E-04 |  | 1.06 | 7.99E-01 |
| B7_3792 | Phenylalanine (phe) | 2.18 | 2.23E-05 |  | 1.71 | 1.64E-03 |  | 1.03 | 8.42E-01 |
| B7_3445 | Phenylalanine (phe) | 2.90 | 4.99E-07 | Phenylalanine (phe) | 2.29 | 2.72E-04 |  | 1.02 | 8.95E-01 |
| B7_3373 | Phenylalanine (phe) | 2.30 | 4.28E-07 |  | 1.98 | 7.44E-06 |  | 1.09 | 4.90E-01 |
| B7_2330 | Unknown | 2.75 | 9.01E-04 |  | 0.87 | 3.22E-01 |  | 0.96 | 7.63E-01 |
| B7_2071 | Tyrosine (tyr) | 6.63 | 8.61E-09 | Tyrosine (tyr) | 4.41 | 1.60E-07 |  | 1.07 | 7.04E-01 |
| B7_2001 | Unknown | 2.52 | 6.72E-06 |  | 1.67 | 1.87E-02 |  | 1.02 | 9.46E-01 |
| B7_1948 | Unknown | 2.74 | 3.48E-05 | Unknown | 2.08 | 4.18E-04 |  | 1.05 | 7.83E-01 |
| B7_1893 | Tyrosine (tyr) | 5.19 | 2.68E-08 | Tyrosine (tyr) | 3.51 | 6.23E-05 |  | 0.97 | 8.91E-01 |
| B6_9132 | Tyrosine (tyr) | 2.91 | 5.59E-07 | Tyrosine (tyr) | 2.22 | 1.77E-05 |  | 1.06 | 5.52E-01 |
| B6_9073 | Unknown | 2.00 | 5.53E-04 |  | 1.97 | 1.09E-04 |  | 1.14 | 5.89E-01 |
| B6_8979 | Unknown | 2.29 | 8.83E-06 |  | 1.83 | 3.23E-04 |  | 1.07 | 6.35E-01 |
| B5_9139 | uridine (uri) | 2.84 | 1.31E-08 |  | 1.07 | 3.86E-01 |  | 1.08 | 5.49E-01 |
| B5_4044 | Unknown | 2.56 | 5.46E-03 | Unknown | 4.50 | 1.96E-02 |  | 1.15 | 6.86E-01 |
| B5_3938 |  | 1.57 | 2.40E-02 | Unknown | 3.17 | 4.01E-03 |  | 0.93 | 8.33E-01 |
| B5_2031 |  | 1.65 | 7.88E-05 | trehalose (tre) | 5.78 | 2.15E-09 |  | 0.89 | 2.26E-01 |
| B5_1931 |  | 1.81 | 3.29E-04 | trehalose (tre) | 5.80 | 1.71E-08 |  | 0.91 | 4.64E-01 |
| B5_1366 |  | 0.83 | 1.95E-01 | Unknown | 7.06 | 3.95E-08 |  | 0.94 | 7.33E-01 |
| B5_1278 |  | 1.06 | 5.05E-01 | Unknown | 8.08 | 6.76E-10 |  | 1.00 | 9.93E-01 |
| B4_9095 |  | 0.54 | 6.40E-04 | Unknown | 2.20 | 9.51E-06 |  | 1.53 | 5.11E-02 |
| B4_9005 |  | 0.71 | 5.21E-02 | Unknown | 3.29 | 3.91E-06 |  | 1.07 | 7.33E-01 |
| B4_6600 | glucose (glc) | 3.23 | 6.06E-04 |  | 1.68 | 1.87E-03 |  | 0.79 | 1.50E-01 |
| B4_6423 | glucose (glc) | 2.87 | 2.93E-04 |  | 1.54 | 3.45E-03 |  | 0.93 | 6.82E-01 |
| B4_5155 |  | 0.50 | 1.47E-01 | Unknown | 2.05 | 3.03E-02 |  | 1.67 | 1.46E-01 |
| B4_5060 |  | 0.33 | 2.06E-02 | Unknown | 2.47 | 6.18E-04 |  | 2.07 | 1.09E-02 |
| B4_4975 |  | 0.35 | 3.65E-02 | Unknown | 3.95 | 2.87E-04 |  | 2.99 | 4.43E-02 |
| B4_4290 |  | 1.04 | 8.31E-01 | Unknown | 3.15 | 2.81E-05 |  | 1.05 | 9.13E-01 |
| B4_4115 |  | 1.16 | 1.48E-01 | Unknown | 6.72 | 9.88E-07 |  | 1.34 | 2.99E-01 |
| B4_2052 |  | 0.52 | 2.75E-02 | Unknown | 2.13 | 7.66E-04 |  | 0.83 | 4.66E-01 |
| B4_1996 |  | 0.72 | 1.16E-01 | Unknown | 2.06 | 1.64E-03 |  | 0.99 | 9.73E-01 |
| B4_1939 | Pyroglutamate (pyroglu) | 3.83 | 8.32E-08 |  | 1.66 | 1.26E-05 |  | 0.84 | 1.68E-01 |
| B4_1815 | Pyroglutamate (pyroglu) | 4.48 | 1.73E-04 |  | 1.00 | 9.92E-01 |  | 0.67 | 3.67E-01 |
| B4_1742 | Pyroglutamate (pyroglu) | 8.76 | 4.38E-09 | 3-hydroxybutyrate (3Hbut) | 2.86 | 8.97E-06 |  | 1.40 | 1.51E-02 |
| B4_1337 | Lactate (lac) | 24.98 | 2.29E-04 | Lactate (lac) | 8.66 | 9.70E-08 |  | 0.92 | 7.33E-01 |
| B4_1199 | Lactate (lac) | 6.21 | 2.36E-10 | Lactate (lac) | 2.20 | 1.61E-05 |  | 0.80 | 8.25E-02 |
| B4_1064 | Unknown | 4.65 | 8.94E-08 | Unknown | 2.01 | 1.44E-04 |  | 0.55 | 2.44E-02 |
| B4_0989 | Unknown | 10.21 | 2.17E-04 | Unknown | 8.00 | 2.48E-03 |  | 1.22 | 9.37E-01 |
| B4_0934 | Lactate (lac) | 3.64 | 5.59E-07 | Lactate (lac) | 2.30 | 3.50E-05 |  | 0.68 | 7.95E-02 |
| B4_0250 | Unknown | 3.81 | 2.03E-06 |  | 0.87 | 8.19E-03 |  | 0.91 | 1.44E-01 |
| B3_9860 | Unknown | 16.61 | 8.24E-07 | Unknown | 8.14 | 5.48E-04 |  | 0.65 | 5.66E-01 |
| B3_9795 | Unknown | 3.12 | 1.25E-06 | Unknown | 2.03 | 1.01E-04 |  | 1.10 | 5.21E-01 |
| B3_9693 | Unknown | 5.08 | 1.01E-04 |  | 1.73 | 1.23E-01 |  | 1.02 | 9.75E-01 |
| B3_9440 | Unknown | 9.25 | 2.99E-07 | Unknown | 2.93 | 5.17E-03 |  | 1.20 | 4.84E-01 |
| B3_8988 |  | 1.20 | 3.13E-01 | Unknown | 5.73 | 8.25E-02 |  | 0.99 | 9.68E-01 |
| B3_8089 | Glycerol | 3.13 | 6.80E-07 |  | 1.47 | 1.68E-03 |  | 1.08 | 7.45E-01 |
| B3_8022 |  | 1.18 | 9.48E-04 |  | 0.65 | 1.19E-07 |  | 0.90 | 1.79E-03 |
| B3_7952 | Glycerol | 22.07 | 9.78E-07 | Unknown | 5.04 | 2.81E-05 |  | 1.25 | 9.09E-02 |
| B3_7375 |  | 0.47 | 8.38E-04 | Unknown | 4.47 | 1.68E-07 |  | 1.06 | 8.73E-01 |
| B3_6983 | Unknown | 3.01 | 1.11E-04 | Unknown | 2.55 | 1.50E-05 |  | 0.89 | 6.71E-01 |
| B3_6702 | Glycerol | 4.24 | 5.72E-08 |  | 1.23 | 1.76E-03 |  | 0.85 | 1.64E-02 |
| B3_6599 | Glycerol | 12.95 | 7.67E-10 |  | 2.00 | 1.72E-04 |  | 1.04 | 7.98E-01 |
| B3_6470 | Glycerol | 15.21 | 2.23E-08 | Trehalose (tre) | 2.02 | 6.76E-10 |  | 0.90 | 3.30E-02 |
| B3_6377 | Glycerol | 31.47 | 1.37E-10 | Trehalose (tre) | 3.25 | 1.74E-08 |  | 1.04 | 6.71E-01 |
| B3_6187 | Valine (val) | 4.19 | 1.82E-07 | Valine (val) | 4.23 | 1.77E-07 |  | 1.09 | 5.89E-01 |
| B3_6124 | Valine (val) | 5.72 | 1.07E-05 | Valine (val) | 3.44 | 2.18E-05 |  | 0.97 | 7.33E-01 |
| B3_5783 | Glycerol | 5.36 | 5.72E-08 |  | 1.01 | 7.56E-01 |  | 1.02 | 8.97E-01 |
| B3_5669 | Glycerol | 15.07 | 2.63E-08 |  | 0.85 | 3.64E-01 |  | 1.27 | 5.24E-01 |
| B3_5630 | Glycerol | 6.39 | 1.31E-08 | Unknown | 2.36 | 7.92E-05 |  | 1.08 | 7.21E-01 |
| B3_5555 | Glycerol | 6.80 | 3.43E-06 |  | 0.58 | 2.00E-01 |  | 0.83 | 7.58E-01 |
| B3_5431 | Glycerol | 6.44 | 1.99E-06 |  | 1.19 | 1.05E-01 |  | 1.09 | 6.79E-01 |
| B3_4226 | Proline (pro) | 13.71 | 4.21E-05 |  | 1.28 | 4.08E-01 |  | 0.71 | 5.01E-01 |
| B3_4193 | Proline (pro) | 2.26 | 2.77E-02 |  | 0.18 | 5.83E-04 |  | 0.99 | 9.75E-01 |
| B3_4151 | Proline (pro) | 4.47 | 3.81E-05 |  | 1.71 | 2.33E-03 |  | 1.00 | 9.86E-01 |
| B3_4019 | Proline (pro) | 9.96 | 1.06E-04 |  | 1.21 | 4.67E-01 |  | 1.04 | 8.95E-01 |
| B3_3678 | Proline (pro) | 4.82 | 1.77E-07 |  | 1.10 | 1.77E-01 |  | 1.06 | 6.20E-01 |
| B3_3430 | Proline (pro) | 2.65 | 1.64E-06 |  | 0.95 | 5.68E-01 |  | 1.04 | 8.42E-01 |
| B3_3379 | Proline (pro) | 5.29 | 1.93E-07 |  | 1.22 | 1.24E-01 |  | 1.07 | 7.33E-01 |
| B3_3306 | Proline (pro) | 2.17 | 6.35E-05 |  | 0.73 | 6.82E-03 |  | 0.94 | 5.21E-01 |
| B3_2978 |  | 1.68 | 2.02E-03 | Unknown | 2.17 | 2.38E-05 |  | 0.95 | 7.63E-01 |
| B3_2354 | Unknown | 2.55 | 3.68E-05 |  | 1.05 | 6.54E-02 |  | 1.04 | 7.16E-01 |
| B3_2209 | Phosphocholine (pchol) | 20.43 | 8.97E-07 |  | 1.65 | 1.29E-02 |  | 1.67 | 5.94E-01 |
| B3_1649 |  | 1.73 | 7.74E-03 | Unknown | 2.10 | 7.18E-03 |  | 0.93 | 8.33E-01 |
| B3_1581 | Ethanolamine (eth) | 4.49 | 5.74E-06 |  | 1.08 | 6.56E-01 |  | 0.89 | 7.17E-01 |
| B3_1470 | Ethanolamine (eth) | 4.02 | 6.01E-05 |  | 1.35 | 9.55E-03 |  | 0.97 | 8.54E-01 |
| B3_1370 | Ethanolamine (eth) | 3.73 | 1.58E-04 |  | 1.84 | 3.71E-03 |  | 1.00 | 9.93E-01 |
| B3_1307 | Ethanolamine (eth) | 2.36 | 3.48E-02 | Unknown | 2.48 | 7.68E-04 |  | 0.87 | 6.32E-01 |
| B3_1115 | Unknown | 3.86 | 3.11E-04 |  | 1.80 | 2.76E-04 |  | 0.91 | 5.17E-01 |
| B3_1040 |  | 1.14 | 4.95E-01 | Unknown | 2.54 | 1.07E-04 |  | 1.03 | 8.95E-01 |
| B3_0771 |  | 1.25 | 1.87E-02 | Unknown | 2.47 | 1.58E-04 |  | 1.20 | 9.15E-02 |
| B3_0517 |  | 0.88 | 1.13E-01 | Ornithine (orn) | 2.77 | 4.97E-04 |  | 1.17 | 3.77E-01 |
| B3_0390 | Unknown | 3.90 | 1.91E-03 | Unknown | 3.28 | 6.62E-03 |  | 0.84 | 7.71E-01 |
| B3_0230 |  | 1.84 | 3.30E-03 | Unknown | 4.13 | 1.67E-04 |  | 1.60 | 8.90E-02 |
| B3_0076 |  | 1.50 | 4.72E-02 | Unknown | 3.68 | 7.10E-04 |  | 1.33 | 2.57E-01 |
| B2_9944 |  | 1.28 | 5.57E-02 | Unknown | 2.85 | 1.77E-03 |  | 0.98 | 9.46E-01 |
| B2_9635 |  | 1.24 | 1.17E-01 | Unknown | 2.73 | 1.83E-04 |  | 1.37 | 9.26E-02 |
| B2_9554 |  | 1.37 | 4.69E-02 | Unknown | 2.84 | 7.90E-05 |  | 1.30 | 2.12E-01 |
| B2_9437 |  | 1.23 | 4.72E-02 | Unknown | 2.33 | 9.54E-05 |  | 1.29 | 5.33E-02 |
| B2_9345 |  | 1.55 | 2.49E-03 | Unknown | 2.49 | 2.92E-05 |  | 1.44 | 2.59E-02 |
| B2_9282 |  | 1.05 | 3.97E-01 | Unknown | 2.12 | 7.59E-04 |  | 1.23 | 3.30E-02 |
| B2_9163 |  | 0.92 | 3.53E-01 | Unknown | 2.07 | 6.95E-05 |  | 1.13 | 4.10E-01 |
| B2_8508 |  | 0.82 | 1.54E-01 | Unknown | 2.90 | 1.01E-04 |  | 0.97 | 9.37E-01 |
| B2_7549 |  | 1.51 | 1.37E-03 | Unknown | 3.21 | 1.83E-04 |  | 1.24 | 8.30E-02 |
| B2_7231 |  | 1.85 | 3.41E-03 | Unknown | 2.04 | 2.63E-03 |  | 1.13 | 6.29E-01 |
| B2_5346 | Pyroglutamate (pyroglu) | 7.26 | 1.88E-05 |  | 1.00 | 9.98E-01 |  | 0.91 | 8.58E-01 |
| B2_5297 | Pyroglutamate (pyroglu) | 9.12 | 1.61E-06 |  | 1.35 | 2.50E-02 |  | 1.46 | 2.52E-02 |
| B2_5163 | Pyroglutamate (pyroglu) | 6.93 | 5.69E-09 |  | 1.07 | 6.13E-01 |  | 1.21 | 1.38E-01 |
| B2_5124 | Pyroglutamate (pyroglu) | 9.00 | 1.10E-06 |  | 1.45 | 1.59E-01 |  | 1.10 | 7.09E-01 |
| B2_5086 | Pyroglutamate (pyroglu) | 2.57 | 6.02E-06 |  | 0.84 | 1.36E-01 |  | 1.08 | 5.89E-01 |
| B2_5035 | Pyroglutamate (pyroglu) | 3.32 | 4.14E-07 |  | 0.85 | 2.11E-01 |  | 1.28 | 4.24E-02 |
| B2_4979 | Pyroglutamate (pyroglu) | 3.69 | 5.21E-07 |  | 1.30 | 8.55E-02 |  | 0.94 | 7.54E-01 |
| B2_4916 | Pyroglutamate (pyroglu) | 3.45 | 4.88E-07 |  | 0.87 | 1.30E-01 |  | 1.09 | 2.97E-01 |
| B2_4720 | Pyroglutamate (pyroglu) | 2.97 | 4.77E-06 |  | 1.12 | 3.10E-01 |  | 0.92 | 5.95E-01 |
| B2_4637 | Pyroglutamate (pyroglu) | 2.76 | 6.72E-06 |  | 1.31 | 3.77E-02 |  | 1.17 | 3.30E-01 |
| B2_4410 | Unknown | 2.32 | 6.35E-05 |  | 0.52 | 1.04E-02 |  | 1.10 | 5.36E-01 |
| B2_4340 |  | 0.39 | 4.08E-02 | 3-hydroxybutyrate (3Hbut) | 4.48 | 2.44E-04 |  | 0.93 | 8.93E-01 |
| B2_4205 | Succinate (succ) | 2.36 | 1.99E-10 |  | 1.82 | 3.65E-09 |  | 1.03 | 8.18E-02 |
| B2_4100 | Pyroglutamate (pyroglu) | 8.54 | 2.23E-06 |  | 1.79 | 3.14E-04 |  | 1.41 | 2.04E-01 |
| B2_4050 | Unknown | 10.93 | 2.37E-08 | 3-hydroxybutyrate (3Hbut) | 3.37 | 4.56E-07 |  | 1.65 | 4.35E-02 |
| B2_4005 | Unknown | 2.18 | 2.25E-05 |  | 0.86 | 4.54E-04 |  | 0.88 | 9.09E-02 |
| B2_3923 | Unknown | 5.87 | 7.05E-08 | Unknown | 4.44 | 5.10E-07 |  | 1.32 | 2.65E-01 |
| B2_3863 | Pyroglutamate (pyroglu) | 9.54 | 2.68E-06 |  | 1.07 | 7.08E-01 |  | 1.81 | 1.08E-01 |
| B2_3766 | Unknown | 2.21 | 1.03E-03 | Glutamate (glu) | 5.72 | 1.13E-05 |  | 1.21 | 4.79E-01 |
| B2_3706 |  | 1.08 | 9.44E-03 | Unknown | 2.08 | 2.60E-04 |  | 0.99 | 7.73E-01 |
| B2_3630 | Unknown | 2.26 | 3.73E-08 | Glutamate (glu) | 4.75 | 6.46E-10 |  | 1.30 | 9.49E-03 |
| B2_3559 |  | 1.97 | 1.07E-04 | Glutamate (glu) | 5.48 | 1.26E-06 |  | 1.44 | 5.73E-04 |
| B2_3460 |  | 1.85 | 4.87E-07 | Glutamate (glu) | 3.59 | 1.64E-08 |  | 1.20 | 5.04E-03 |
| B2_3404 |  | 1.48 | 1.85E-05 | Glutamate (glu) | 5.28 | 6.46E-10 |  | 1.33 | 2.76E-03 |
| B2_3328 | Unknown | 2.16 | 6.72E-06 | Unknown | 3.85 | 2.92E-04 |  | 1.20 | 8.52E-02 |
| B2_3260 |  | 0.94 | 5.92E-01 | Unknown | 2.40 | 7.49E-03 |  | 1.34 | 3.69E-02 |
| B2_3166 |  | 1.48 | 6.66E-05 | Unknown | 2.87 | 1.94E-04 |  | 1.13 | 1.18E-01 |
| B2_2832 | Unknown | 2.42 | 9.70E-04 | Unknown | 2.81 | 9.11E-05 |  | 0.81 | 3.36E-01 |
| B2_2610 | Unknown | 5.67 | 1.38E-06 | Unknown | 2.77 | 3.05E-04 |  | 1.02 | 9.50E-01 |
| B2_2394 | Unknown | 26.88 | 6.18E-11 | Unknown | 2.01 | 1.15E-02 |  | 1.18 | 7.18E-01 |
| B2_1281 |  | 1.55 | 1.38E-01 | Glutamate (glu) | 6.19 | 1.10E-05 |  | 0.79 | 6.71E-01 |
| B2_0976 | Proline (pro) | 4.05 | 1.12E-06 | Glutamate (glu) | 3.57 | 9.28E-06 |  | 1.02 | 9.50E-01 |
| B2_0833 | Proline (pro) | 3.41 | 2.37E-08 | Glutamate (glu) | 4.78 | 9.06E-10 |  | 1.24 | 1.61E-02 |
| B2_0740 | Proline (pro) Pyroglutamate (pyroglu) | 6.13 | 2.70E-06 |  | 1.09 | 6.88E-01 |  | 1.00 | 9.93E-01 |
| B2_0672 | Proline (pro) Pyroglutamate (pyroglu) | 2.72 | 4.50E-08 | Glutamate (glu) | 4.76 | 7.03E-10 |  | 1.25 | 5.77E-03 |
| B2_0614 | Proline (pro) Pyroglutamate (pyroglu) | 14.57 | 7.49E-07 |  | 1.32 | 1.66E-01 |  | 1.30 | 5.89E-01 |
| B2_0577 | Proline (pro) Pyroglutamate (pyroglu) | 10.34 | 1.31E-08 |  | 1.49 | 2.94E-02 |  | 0.93 | 8.42E-01 |
| B2_0525 | Proline (pro) Pyroglutamate (pyroglu) | 2.94 | 7.25E-06 | Glutamate (glu) | 4.79 | 1.00E-06 |  | 1.24 | 2.30E-02 |
| B2_0472 | Proline (pro) Pyroglutamate (pyroglu) | 7.75 | 2.10E-08 |  | 1.42 | 3.00E-02 |  | 1.01 | 9.50E-01 |
| B2_0431 | Proline (pro) Pyroglutamate (pyroglu) | 10.92 | 5.72E-08 | Glutamate (glu) | 2.60 | 3.45E-04 |  | 0.88 | 7.10E-01 |
| B2_0397 | Proline (pro) Pyroglutamate (pyroglu) | 3.46 | 8.61E-07 | Glutamate (glu) | 3.90 | 8.98E-07 |  | 1.24 | 4.75E-02 |
| B2_0356 | Proline (pro) Pyroglutamate (pyroglu) | 3.43 | 1.16E-06 | Glutamate (glu) | 4.42 | 6.31E-07 |  | 1.18 | 1.60E-01 |
| B2_0294 | Proline (pro) Pyroglutamate (pyroglu) | 7.72 | 4.99E-08 |  | 1.41 | 6.42E-03 |  | 1.08 | 4.27E-01 |
| B2_0181 | Proline (pro) Pyroglutamate (pyroglu) | 4.04 | 6.84E-06 |  | 1.57 | 3.90E-03 |  | 1.06 | 5.13E-01 |
| B2_0055 | Proline (pro) Pyroglutamate (pyroglu) | 3.32 | 1.99E-10 |  | 1.21 | 3.36E-02 |  | 0.99 | 8.76E-01 |
| B1_9910 | Proline (pro) Pyroglutamate (pyroglu) | 4.08 | 1.14E-05 |  | 1.70 | 1.33E-03 |  | 1.13 | 5.89E-01 |
| B1_9245 |  | 1.06 | 8.47E-01 | Unknown | 2.41 | 9.04E-03 |  | 0.46 | 2.84E-01 |
| B1_6695 | Unknown | 2.19 | 8.13E-03 |  | 1.91 | 2.24E-02 |  | 1.07 | 8.95E-01 |
| B1_5288 |  | 0.37 | 2.27E-02 | Unknown | 2.04 | 1.15E-02 |  | 1.50 | 2.04E-01 |
| B1_4906 | Alanine (ala) | 4.78 | 1.26E-10 | Alanine (ala) | 3.23 | 6.76E-10 |  | 1.33 | 3.18E-04 |
| B1_4768 | Alanine (ala) | 5.29 | 1.44E-07 | Alanine (ala) | 3.44 | 9.06E-10 |  | 1.38 | 3.18E-04 |
| B1_3583 | Unknown | 2.29 | 4.88E-05 |  | 1.36 | 2.23E-02 |  | 1.21 | 4.27E-01 |
| B1_3360 | Lactate (lac) | 3.60 | 6.00E-10 | Lactate (lac) | 2.27 | 2.44E-07 |  | 0.91 | 2.02E-01 |
| B1_3211 | Lactate (lac) | 4.09 | 6.51E-10 | Lactate (lac) | 2.54 | 4.82E-08 |  | 0.89 | 2.02E-01 |
| B1_2099 |  | 0.98 | 9.37E-01 | 3-hydroxybutyrate (3Hbut) | 21.86 | 1.18E-09 |  | 0.95 | 8.81E-01 |
| B1_1969 |  | 0.65 | 5.89E-02 | 3-hydroxybutyrate (3Hbut) | 9.11 | 4.70E-06 |  | 0.93 | 5.54E-01 |
| B1_0930 | Unknown | 2,50 | 1,11E-04 |  | 0,95 | 6,51E-01 |  | 1,26 | 1,73E-01 |
| B1_0799 | Unknown | 3,22 | 1,87E-05 |  | 1,19 | 2,00E-01 |  | 1,07 | 8,10E-01 |
| B1_0540 | Valine (val) | 3.95 | 3.93E-07 | Valine (val) | 3.77 | 5.76E-07 |  | 1.07 | 4.31E-01 |
| B1_0360 | Valine (val) | 3.86 | 6.80E-07 | Valine (val) | 3.61 | 1.46E-07 |  | 1.14 | 9.88E-02 |
| B1_0207 | Isoleucine (ile) | 3.83 | 1.31E-08 | Isoleucine (ile) | 3.81 | 8.72E-09 |  | 1.05 | 5.15E-01 |
| B1_0096 | Isoleucine (ile) | 5.34 | 2.04E-06 | Isoleucine (ile) | 5.61 | 1.33E-07 |  | 1.26 | 3.30E-02 |
| B1_0004 | Valine (val) | 7.15 | 6.57E-08 | Valine (val) | 6.53 | 2.20E-07 |  | 1.33 | 1.51E-02 |
| B0_9890 | Valine (val) | 6.38 | 8.35E-10 | Valine (val) | 5.48 | 6.76E-10 |  | 1.19 | 2.59E-02 |
| B0_9830 | Unknown | 5.18 | 7.44E-05 | Unknown | 6.10 | 2.81E-05 |  | 0.98 | 9.88E-01 |
| B0_9750 | Leucine (leu) | 4.28 | 3.71E-05 | Leucine (leu) | 3.31 | 6.33E-07 |  | 0.89 | 3.09E-01 |
| B0_9642 | Leucine (leu) | 4.29 | 1.99E-10 | Leucine (leu) | 3.65 | 1.77E-07 |  | 0.93 | 4.84E-01 |
| B0_9574 | Isoleucine (ile) | 7.92 | 3.97E-06 | Isoleucine (ile) | 7.43 | 4.38E-08 |  | 1.20 | 4.09E-01 |
| B0_9511 | Leucine (leu) | 5.88 | 8.35E-08 | Leucine (leu) | 6.11 | 2.88E-06 |  | 1.04 | 8.68E-01 |
| B0_9430 | Isoleucine (ile) | 4.01 | 3.64E-09 | Isoleucine (ile) | 4.45 | 3.72E-07 |  | 0.90 | 2.05E-01 |
| B0_9268 | Isoleucine (ile) | 5.96 | 2.37E-08 | Isoleucine (ile) | 7.79 | 4.90E-07 |  | 1.09 | 5.94E-01 |
| B0_8998 |  | 0.49 | 2.08E-03 | Unknown | 2.16 | 2.66E-05 |  | 0.87 | 7.33E-01 |

**Online Resource 4:** NMR annotation table of compounds in feed extracts that fulfil volcano plot thresholds.

| Compounds | CHEBI | ^1^H detected (ppm) | J (Hz) | COSY  (^1^H-^1^H) | HSQC  (^1^H-^13^C) | HMBC  (^1^H-^13^C) | MSI level |
| --- | --- | --- | --- | --- | --- | --- | --- |
| Isoleucine | 24898 | 0.94 (t) | 7.3 |  | 13.9 |  | 1 |
|  |  | 1.01 (d) | 7.2 |  | 17.7 | 62.4, 40 |  |
| Leucine | 25017 | 0.96 (t) | 5.73 | 1.71 | 23.8, 24.9 | 27.06 | 1 |
| Valine | 27266 | 0.99 (d), 1.04 (d) | 7.04 | 2.28, 3.60 | 19.53, 20.74 | 19.6, 20.8 32.1, 63.3 | 1 |
|  |  | 3.61 (d) |  |  | 69.23 |  |  |
| 3-hydroxybutyrate | 37054 | 1.20 (d) | 6.24 | 4.1 | 24.55 | 22.4, 23.0, 49.5, 68.6 | 1 (spiked compound) |
|  |  | 4.17 (m) |  |  |  |  |  |
| Lactate | 24996 | 1.33 (d) | 6.97 | 4.11 | 22.93 | 71.3 | 1 |
|  |  | 4.12 (q) | 6.90 | 1.32 | 71.37 |  |  |
| Alanine | 16449 | 1.48 (d) | 7.30 | 3.80 | 18.89 | 53.3, 178.7 | 1 |
| Proline | 26271 | 2.05 (m) |  |  |  |  | 1 (spiked compound) |
|  |  | 2.35 (m) |  |  |  |  |  |
|  |  | 3.38 (m) |  |  | 48.96 |  |  |
|  |  | 4.13 (dd) |  |  | 64.07 |  |  |
| Pyroglutamate | 57606 | 2.04 (m) |  |  |  |  | 1 (spiked compound) |
|  |  | 2.41 (m) |  |  |  |  |  |
|  |  | 2.50 (m) |  |  |  |  |  |
|  |  | 4.19 (dd) | 8.84, 5.40 |  |  |  |  |
| Glutamate | 29987 | 2.08 (m) |  |  |  |  | 1 (spiked compound) |
|  |  | 2.36 (m) |  |  |  |  |  |
| Succinate | 26806 | 2.42 (s) |  |  | 36.7 |  | 1 (spiked compound) |
| Ornithine | 18257 | 3.06 (t) |  |  |  |  | 1 (spiked compound) |
| Ethanolamine | 16000 | 3.14 (t) | 5.30 | 3.84 | 44.13 |  | 1 |
| Phosphocholine | 18132 | 3.22 (s) |  |  | 56.8 | 68.5 | 1 (spiked compound) |
| Carnitine | 17126 | 3.23 (s) |  |  |  |  | 1 (spiked compound) |
| Glycerol | 17754 | 3.56 (m) |  | 3.65, 3.78 | 65.43 | 65.37, 74.94 | 1 |
|  |  | 3.65 (m) |  | 3.55, 3.78 | 65.38 | 65.37, 74.94 |  |
|  |  | 3.79 (tt) | 4.40 | 3.55, 3.65 | 74.96 | 65.35 |  |
| Trehalose | 16551 | 3.45 (t) | 9.80 |  |  |  | 1 |
|  |  | 3.63 (dd) | 3.81, 9.94 | 5.20 | 73.91 |  |  |
|  |  | 5.20 (d) | 3.80 | 3.60 | 96.11 |  |  |
| Glucose | 17234 | 3.4 (m) |  |  |  |  | 1 |
|  |  | 4.65 (d) | 7.95 | 3.24 | 98.8 |  |  |
|  |  | 5.23 (d) | 3.51 | 3.56 | 94.9 |  |  |
| Uridine | 16704 | 5.91 (m) |  |  |  |  | 1 (spiked compound) |
|  |  | 7.87 (d) |  |  |  |  |  |
| Tyrosine | 18186 | 6.91 (m) |  | 7.19 | 118.63 |  | 1 |
|  |  | 7.20 (m) |  | 6.90 | 133.59 |  |  |
| Phenylalanine | 28044 | 7.31 (d) | 7.42 | 7.42 | 132.6 |  | 1 |
|  |  | 7.41 (m) |  | 7.33 | 131.95 |  |  |
| Hypoxanthine | 17368 | 8.19 (s) |  |  |  |  | 1 (spiked compound) |
|  |  | 8.21 (s) |  |  |  |  |  |
| AMP | 16027 | 8.27 (s) |  |  |  |  | 1 (spiked compound) |

**Online Resource 5.** Molecular weight profile of insect meal

| PM > 35000 | 0.65 |
| --- | --- |
| 35000 > PM > 18000 | 2.12 |
| 18000 > PM > 10000 | 4.05 |
| 10000 > PM > 6000 | 4.48 |
| 6000 > PM > 3000 | 9.08 |
| 3000 > PM > 2500 | 3.53 |
| 2500 > PM > 2000 | 4.71 |
| 2000 > PM > 1500 | 7.76 |
| 1500 > PM > 1000 | 13.66 |
| 1000 > PM > 800 | 10.06 |
| 800 > PM > 500 | 12.18 |
| 500 > PM > 204 | 21.95 |
| 204 > PM | 5.77 |
| Mean molecular weight | 2820 |
| Peptide fraction < 1000 Da | 49.96 |

**Online Resource 6.** Zoom in NMR spectra of insect and spirulina diet and feedstuff stacked with corresponding standard compound identified in diet. A) Spectral region of glycerol resonances (δ 3.4-3.9 ppm) in INS diet, feedstuff and in standard compound spectra; B) Spectral region of 3-hydroxybutyrate resonances (δ 1.17-1.24 ppm; δ 2.30-2.44 ppm and δ 4.10-4.19 ppm) in SPI diet, feedstuff and in standard compound.


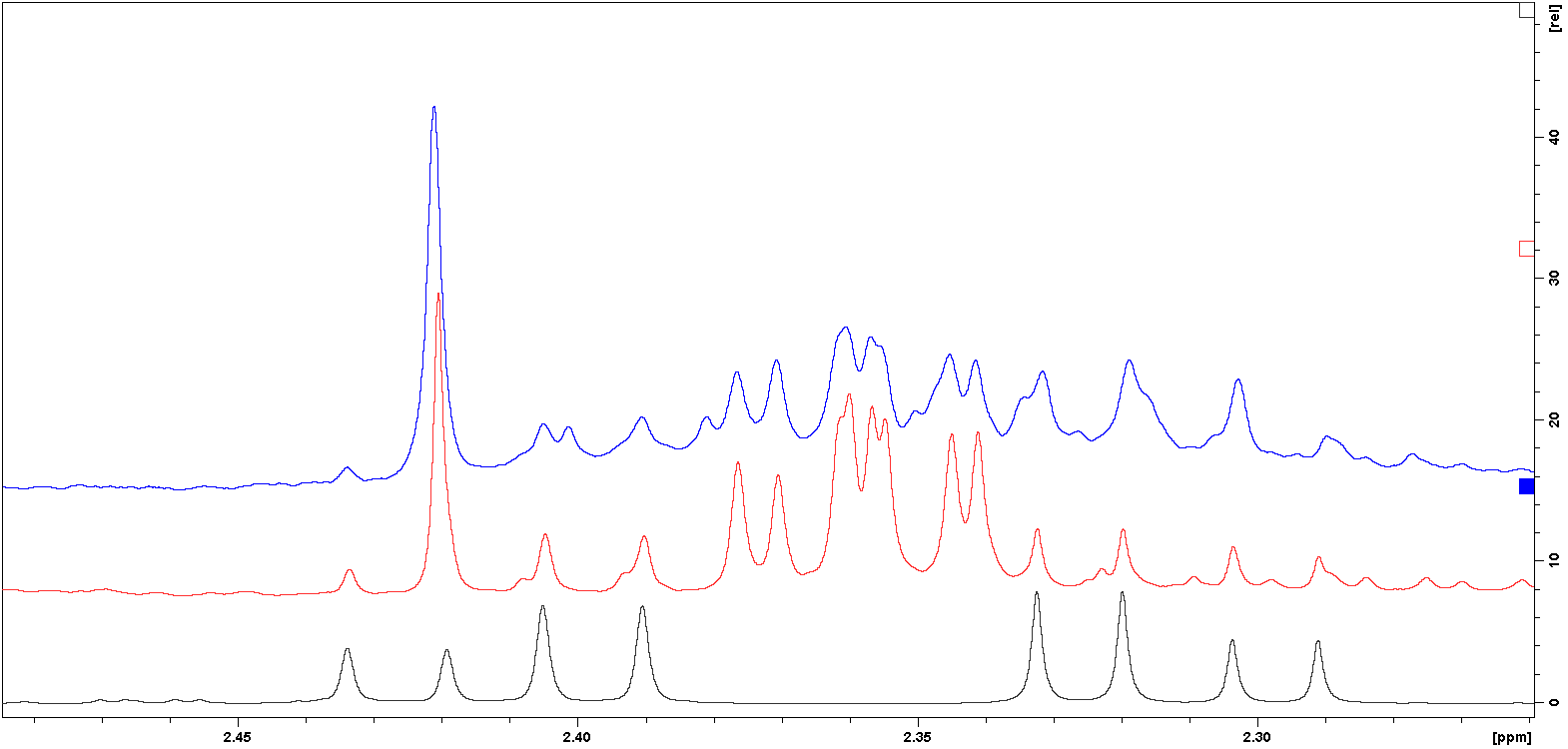

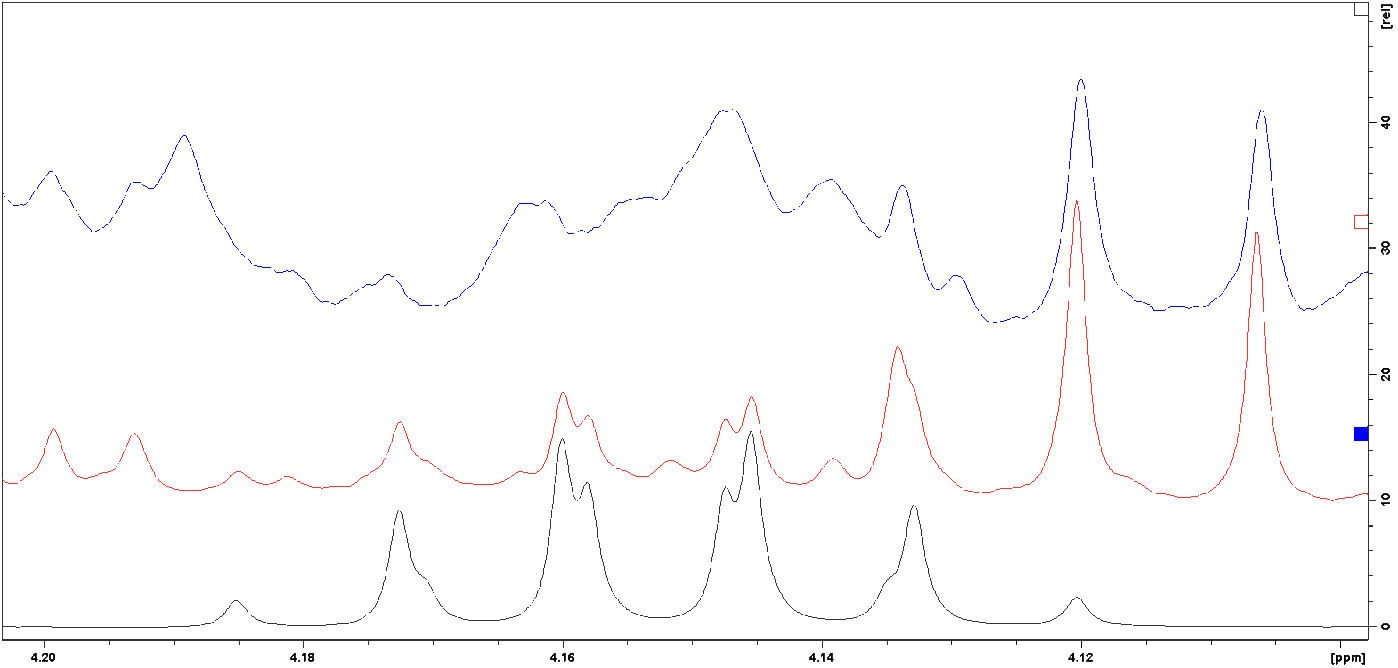

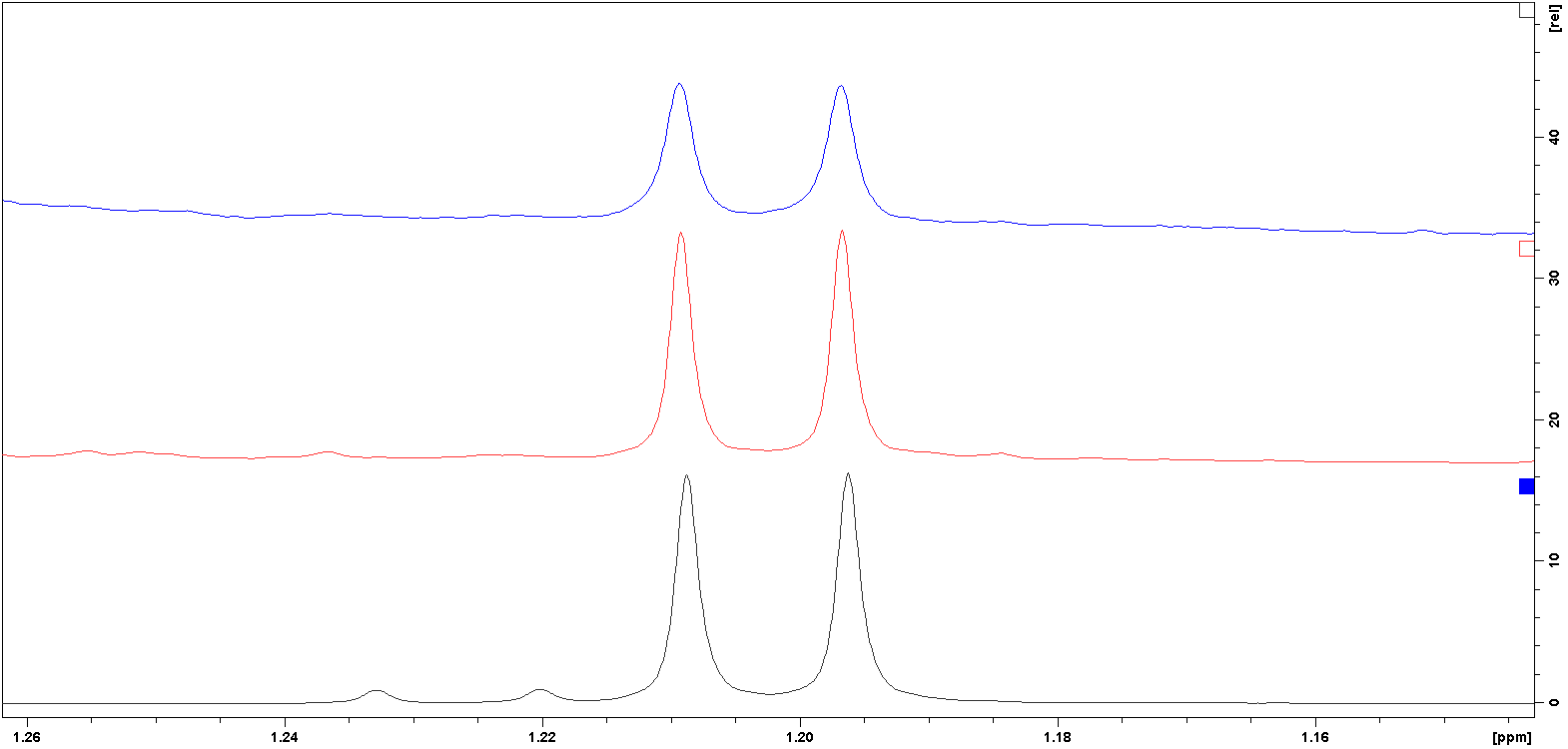


SPI diet

SPI feedstuff

3-hydroxybutyrate standard compound

**B**


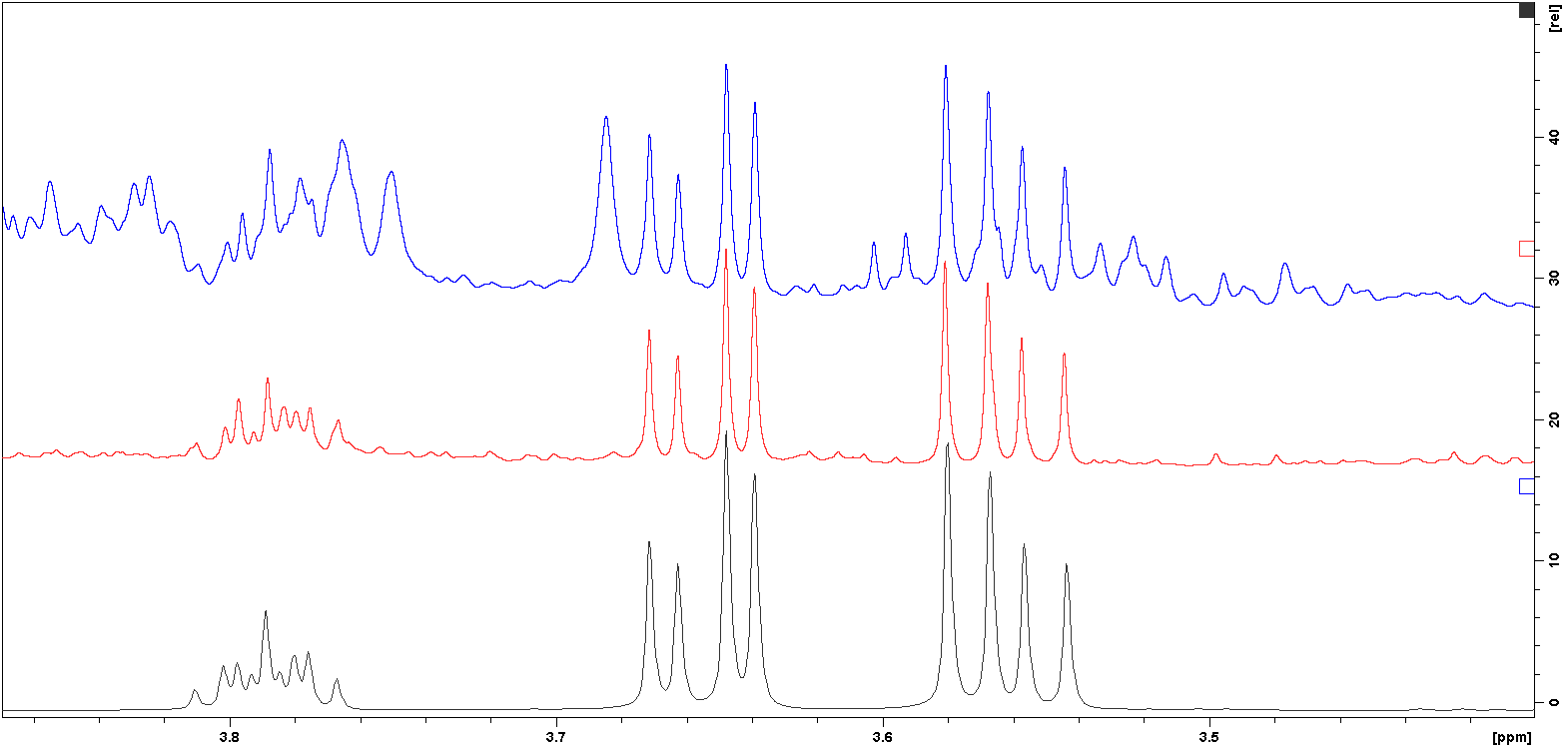


INS diet

INS feedstuff

glycerol standard compound

**A**
